# Impacts of environmental matching on the routine metabolic rate and mass of native and mixed-ancestry brook trout (*Salvelinus fontinalis*) fry

**DOI:** 10.1093/conphys/coy023

**Published:** 2018-05-08

**Authors:** Catharine J Cook, Chris C Wilson, Gary Burness

**Affiliations:** 1 Environmental and Life Sciences Graduate Program, Trent University, Peterborough, ON, Canada K9L 0G2; 2 Aquatic Research and Monitoring Section, Ontario Ministry of Natural Resources and Forestry, Trent University, Peterborough, ON, Canada K9L 0G2; 3 Department of Biology, Trent University, Peterborough, ON, Canada K9L 0G2

**Keywords:** Climate change, epigenetics fish, physiology, temperature

## Abstract

The environment an organism experiences during early development can impact its physiology and survival later in life. The objective of this study was to determine if temperatures experienced at embryonic life stages of brook trout (*Salvelinus fontinalis*) affected mass and routine metabolic rate (RMR) of a subsequent life stage (free-swimming fry). As part of this, we assessed the contributions and importance of hierarchical levels of biological organization [ancestral type (native vs. hatchery-introgressed), population, and family] to variability in mass and RMR of fry. As embryos and alevin, individuals were reared at either natural environmental (5°C) or elevated (9°C) temperatures and then acclimated to either matched or mismatched temperature treatments once yolk sacs were resorbed. Mass differences among fry were strongly influenced by population of origin as well as initial rearing and final acclimation temperatures. Variation in mass-adjusted RMR of fry was also strongly accounted for by source population, acclimation temperature, and individual mass. A significant interaction between population RMR and final acclimation temperature indicated that not all brook trout populations responded the same way to temperature changes. In contrast to expectations, the highest ancestry category (native vs. introgressed) did not significantly influence mass or mass-adjusted RMR.

## Teaser

Rearing environments can have long-term effects on individuals. We tested whether incubation temperatures experienced by brook trout embryos would have carry-over effects on the metabolic rates of free-swimming fry. Although metabolism differed among populations, we found no evidence that temperature at one life-stage influenced metabolism in a subsequent life stage.

## Introduction

Environmental temperature is a critical factor that contributes to species’ distributions and abundances, in large part due to its effect on physiological processes (Levins, 1968; Anguilleta, 2009; Schulte *et al.*, 2011). With increasing temperatures, performance traits increase until fitness/performance is maximized; although temperatures beyond this optimum result in performance declines and reduced scope for activity (Anguilleta, 2009; Schulte *et al.*, 2011). Increasing global temperatures are contributing to concerns about the capacity for many species and populations to tolerate or adapt to rising temperatures, with resultant conservation implications (Carroll *et al.*, 2014). This is particularly true for freshwater species that are unable to relocate to more favourable environments without direct, suitable habitat connections (McCullough *et al.*, 2009; Whitney *et al.*, 2016).

There is particular concern about the future of coldwater fishes in the face of climatic warming (Flebbe *et al.*, 2006; McCullough *et al.*, 2009; Whitney *et al.*, 2016). Studies on an array of coldwater fishes have explored the impacts of rising water temperatures on various physiological traits, including oxygen consumption rate (eelpout, *Zoarces viviparous*, Pörtner and Knust, 2007; lake whitefish, *Coregonus clupeaformis*, Mueller *et al.*, 2015), scope for activity (lake trout, *Salvelinus namaycush*, Kelly *et al.*, 2014; brook trout, *S. fontinalis*, Stitt *et al.*, 2014; sockeye salmon, *Oncorhynchus nerka*, Eliason *et al.*, 2011), growth rates (brook trout, Başçinar and Okumuş, 2002), and cellular-level stress (Chadwick *et al.*, 2015; Chadwick and McCormick, 2017). With an inability to migrate beyond local watersheds, stenothermal species may benefit from management techniques such as assisted migration (Aitken and Whitlock, 2013; Carlson *et al.*, 2014), whereby individuals adapted to warmer waters are used to supplement populations that display lower thermal tolerance, or by reintroduction to areas in which they have been extirpated. If adequate genotypic variation exists within the source population, assisted migration may enable accelerated adaptation in the direction in which selective pressures are already occurring (Aitken and Whitlock, 2013). Assisted gene flow between populations with differing adaptations also carries inherent risks, however, and should be evaluated thoroughly before adopting evolutionary rescue as a management tool (Garant *et al.*, 2007).

Early developmental life stages of fish are particularly susceptible to environmental temperature variation, as individuals have limited to no mobility (Fry *et al.*, 1946). Recent evidence suggests that environments experienced at one life stage can have carry-over effects on subsequent life stages, impacting an individual’s subsequent phenotype as well as potential survival (Harvey, 2012; Jonsson and Jonsson, 2014; Mueller *et al.*, 2015). Interactions between developmental conditions and acclimation capacity/thermal tolerance later in life have been studied in a handful of aquatic fishes, including zebrafish (*Danio rerio*; Schaefer and Ryan, 2006; Scott and Johnston, 2012; Schnurr *et al.*, 2014) and mosquitofish (*Gambusia holbrooki*; Seebacher *et al.*, 2014; Ghanizadeh Kazerouni *et al.*, 2016). An emerging pattern is that individuals have highest performance when tested under environmental conditions most closely matching those in which they had previously been raised (Scott and Johnston, 2012; Jonsson and Jonsson, 2014).

As a stenothermal, cold-adapted species, brook trout are well-suited for temperature-related studies (Fry *et al.*, 1946; Balon, 1980; Chadwick *et al.*, 2015). Brook trout are native to eastern North America, and occur in cool, well-oxygenated streams and lakes, and are largely limited to stream networks across most of their range (Scott and Crossman, 1998). Spawning occurs in the fall in shallow areas near groundwater upwellings (~4–5°C) with rocky substrate (Power, 1980; Curry and Noakes, 1995; Ridgway and Blanchfield, 1998; Franssen *et al.*, 2013). Alevin hatch over the winter and remain in redds until their yolk sac is absorbed (May/June) whereupon they move to warmer nearshore waters where food is more abundant (Biro, 1998). After a brief interval in this warm productive habitat, the young brook trout move into cooler, well-oxygenated water, either upstream or into deeper lake waters (Power, 1980; Scott and Crossman, 1998). With the dependence of brook trout on coldwater habitats, climate-change-related stressors have been identified as significant concerns for population sustainability, particularly at the southern range margins (Meisner, 1990; Flebbe *et al.*, 2006).

Physiological traits vary at many levels of biological organization, including ancestry (e.g. native or hatchery introgressed; Danzmann and Ihssen, 1995; Danzmann *et al.*, 1998; McDermid *et al.*, 2012), population (Rieman *et al.*, 2007; Kelly *et al.*, 2014) and even family of origin (Pakkasmaa *et al.*, 2006; Burt *et al.*, 2011; McDermid *et al.*, 2012). Recently, we showed that the level of biological organization contributing most to variation in routine metabolic rate of brook trout (RMR) changed with increasing organismal complexity from egg to alevin (Cook *et al.*, 2018). At the earliest life stages, family and population were the only significant factors contributing to variation in RMR; in subsequent life stages mass and temperature effects assumed increased importance in addition to family or population (Cook *et al.*, 2018). Although we found that ancestry (native versus introgressed) did not have a significant effect on RMR under stable environmental conditions, the impact of ancestry on an individual’s response to changing conditions during development remains unknown.

Hatchery strains of brook trout with ancestry from more southern (US) populations have relatively higher thermal tolerance, and differ in their metabolic responses to rising temperatures, than more northern strains and populations (McDermid *et al.*, 2012; Stitt *et al.*, 2014). If phenotypic variation in mass and RMR are influenced by ancestry, then wild populations with native versus introgressed ancestry may show differential response to matched or mismatched conditions during development, depending on the direction of temperature change. Understanding the impact of differing environmental temperatures across life stages on growth rates and energy expenditure of juveniles can inform management decisions surrounding recovery efforts, such as the potential benefits and choice of populations for assisted migration.

The objective of the current study was to explore the impact of environmental temperature during early development on the subsequent RMR of brook trout fry when matched or mismatched to the temperatures they experienced during early development. We studied four populations; two wild, historically stocked populations with mixed ancestry (introgressed with fish from the more southern ancestry strain used by Stitt *et al.*, 2014), and two wild populations with known native ancestry (Al-Shamlih, 2013; Harbicht *et al.*, 2014). We therefore predicted that individuals from these introgressed populations would perform better (i.e. have greater mass and lower RMR as fry) when maintained at constant warmer temperatures. In contrast, we predicted that individuals from native ancestry populations would perform better when switched from cold temperatures during egg incubation to warmer temperatures during the fry stage, a change in temperature designed to mimic that experienced by wild northern populations at fry emergence (Biro, 1998).

## Methodology

Experiments were carried out on wild-origin egg families from four wild brook trout populations in Algonquin Park, Ontario, Canada, differing in ancestry. For the purpose of this study, ‘ancestry’ was divided into two categories and defined as wild fish with either native (Charles Lake and Dickson Lake) or hatchery introgressed origins (Scott Lake and Stringer Lake). Although Charles Lake has been stocked in the past with the Hills Lake hatchery strain (Ontario Ministry of Natural Resources and Forestry (OMNRF), unpubl. data), brook trout from this lake show no evidence of introgression and have maintained their in this native genetic ancestry (Al-Shamlih, 2013). ‘Populations’ were defined as sets of individuals originating from different wild sources or lakes; ‘families’ in this study were sets of offspring spawned from a single male and female from the same population.

Wild spawn collections and mating designs have been described previously (Cook *et al.*, 2018). Briefly, from each of the four populations, six families were founded from wild single-pair crosses (one male and one female parent), with each adult used only once. Eggs were dry stripped from females and fertilized with milt which was initiated by adding lake water to the jars. Fertilized eggs were kept refrigerated overnight (4°C) at a field camp before being transported to the OMNRF Codrington Fisheries Research Facility (Codrington, Ontario; 44°08’49”N, 77°48’10”W) for rearing. Parent fish were sampled for genetic analysis prior to release by taking nonlethal finclips (≤0.25 cm^2^), which were dried in scale envelopes and stored at room temperature until subsequent DNA extraction could occur (see below).

**Figure 1:**
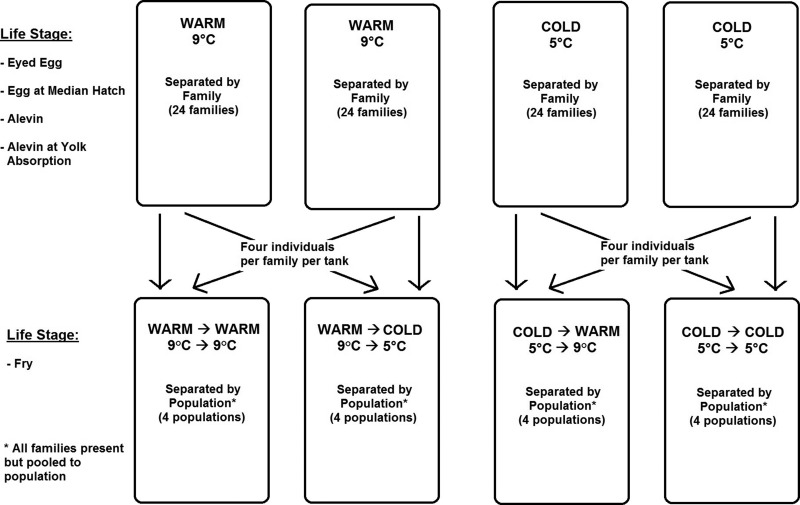
Experimental design aimed at identifying the effects of matched or mismatched environmental temperatures between life stages, on the resultant size and metabolic rates of brook trout fry.

Individuals were reared at either 5°C (cold) or 9°C (warm) as embryos and alevin in replicate acclimation tanks (details in Cook *et al.*, 2018). These temperatures represent those experienced by brook trout embryos in the wild (5°C), and their upper thermal limit (9°C; Curry and Noakes, 1995). Once yolk sacs were absorbed (end of the alevin life stage), fry were moved to create a temperature matched/mismatched design (described below) to examine carry-over effects of early developmental acclimation to one temperature and their metabolic response to the same or a different temperature later in life (Fig. 1). At yolk absorption, eight fry from each family (four from each of the two acclimation tanks held at 5°C or 9°C) were transferred out of the egg boxes and into larger self-cleaning boxes (24 cm × 25 cm × 28 cm) of the same temperature as previous rearing. Four boxes per tank (same tanks as used previously) kept fry separated by population; families from within each population were pooled due to growing requirements and space constraints, and subsequently identified to family using genotyping and parentage analysis (described below). In order to create the four treatment tanks for the fry life stage testing, fry were transferred to three additional self-cleaning boxes following the above procedure making a total of four identical tank set ups (Fig. 1). Once all fry were transferred to the self-cleaning boxes in new 200 l acclimation tanks, they were given food (EWOS #0 MicroCrumble) and fed twice daily to satiation. It took ~1 week before the fry in all tanks were feeding, at which point two of these tanks were either increased or decreased by 1°C per day to the other study temperature (5°C or 9°C) creating the four matched or mismatched treatments (Fig. 1); (i) continued rearing at 9°C (warm–warm), (ii) warm egg/alevin incubation temperature with cold feeding temperature (warm–cold), (iii) cold early rearing temperature with warm feeding temperature (cold–warm) and (iv) continued rearing at 5°C (cold–cold). Fry were left to acclimate to these conditions for 30 days.

### Routine metabolic rate

RMR was measured in fry one month post yolk absorption, following the 30-day acclimation period to either 5°C or 9°C (see Table 1 for degree day ages; Fig. S1 for fry growth rates). Individuals were deprived of food for 2 days prior to testing (Scott and Johnston, 2012). The measurement of RMR occurred in a closed respirometry system using 12 identical glass chambers (described in Cook *et al.*, 2018). Briefly, chambers had an inner compartment (20 ml) containing a single fry and an outer compartment (400 ml) through which freshwater flowed, allowing the temperature of the chamber to be regulated. The inner compartment contained a mini-stir bar, and the chamber was placed on a stir plate to ensure that during measurement water moved past the oxygen probe (Vernier Technologies S120, Beaverton, OR, USA). The mini-stir bar was covered with a screen mesh to prevent contact with the fry. Fry were acclimated for 60 min to the chambers, at which point dissolved oxygen (DO) probes were inserted into the chamber and testing began. Measurement of oxygen consumption rate continued for 90 min or until DO reached a pre-determined limit of 3.5 mg l^−1^, well above the lethal limit of 1.25 mg l^−1^ for brook trout (Graham, 1949; Shepard, 1955). Although oxygen was maintained above lethal limits, we do not know whether any metabolic suppression may have occurred as a result of declining oxygen levels within the chambers. Once a trial ended, fry were removed and chambers were immediately sealed again for 60 min to measure microbial oxygen consumption, which was subtracted from the original consumption of the fish. Following measurement of RMR, fry were euthanized with an overdose of MS-222, patted dry with paper towels, and weighed before being placed in labelled micro-centrifuge tubes and stored at –80°C for genotyping.

**Table 1:** Age of brook trout fry (in degree days, 30 calendar days post yolk absorption) at which routine metabolic rate measurements were performed within the four treatment groups

Temperature treatment^a^	Age in degree days (average ± SE)^b^	Sample size
(i) Warm–Warm (9°C → 9°C)	1133 ± 1.20	157
(ii) Warm–Cold (9°C → 5°C)	1015 ± 1.54	146
(iii) Cold–Warm (5°C → 9°C)	1172 ± 1.42	132
(iv) Cold–Cold (5°C → 5°C)	1065 ± 1.39	110

^a^Individuals were reared at: (i) entire life at 9°C, (ii) eyed egg to yolk absorption at 9°C, then fry at 5°C, (iii) eyed egg to yolk absorption at 5°C, then fry at 9°C, and (iv) entire life at 5°C.

^b^Degree days were calculated by summing the average daily temperature above 0°C experienced by the developing eggs/alevin/fry.

### Genotype analysis

To assign individuals to families, genomic DNA was extracted from adult fin clips and offspring (fry) tail tissue using a simplified protocol for salmonid fishes (Taggart *et al.*, 1992). Parents and offspring were genotyped for 14 microsatellite loci [*Sfo*12, *Sfo*18, *Sfo*23 (Angers *et al.*, 1995), *Sfo*B52, *Sfo*C24, *Sfo*C28, *Sfo*C38, *Sfo*C86, *Sfo*C88, *Sfo*C113, *Sfo*C115, *Sfo*C129, *Sfo*D75, *Sfo*D100 (King *et al.*, 2012)]. Multilocus genotypes were amplified in four multiplex polymerase chain reaction (PCR) reactions, following conditions described by King *et al.* (2012). PCR products were run on an ABI 3730 automated DNA sequencer (Life Technologies Inc.) and scored using GeneMapper version 4.0 software (Applied Biosystems Inc.) and manual proofreading.

Genotypes of fry from the pooled (within-population) families were identified to family using parentage analysis. Fry genotypes were tested against those from candidate parents (mated pairs) using Whichparents software version 1.0 (Hedgecock and Eichert, 1999), which uses parent–offspring matching or exclusion based on multilocus genotypes. This programme was used instead of other parentage analysis software packages due to its ability to use known matings (closed system) to predict offspring assignments to families (Jones *et al.*, 2010). As all offspring were derived from known monogamous (single-pair) matings within each source population, offspring were assigned to candidate parent pairs using exclusion based on multilocus genotypes. Two rounds of analyses were run to assign offspring to families: the first assumed zero error rate for genotyping and scoring (no mismatches between parent and offspring genotypes), the second allowed for one mismatch (one differing allele across the 14 microsatellite loci) between juveniles and candidate parents. Family assignment was used for all subsequent statistical analyses.

### Statistical analysis

To understand the major sources of variation in mass and RMR, we used an information theoretic approach, corrected for small sample sizes (AICc; Burnham and Anderson, 2002). We used a generalized linear mixed model (GLMM) at the fry life stage, and tested for effects of ancestry (native vs. introgressed), population (Charles, Dickson, Scott and Stringer Lakes) and family, as well as temperature (rearing and measurement temperature), and mass as a covariate (RMR analysis only) with two-way interactions. Temperature was separated into two main effects: initial rearing temperature (Temp_Init_) and the post-absorptive acclimation temperature (Temp_Final_). Twenty-five and 54 candidate models were selected for analysis of mass and RMR, respectively, based on *a priori* knowledge of the working system and included all single parameter models (for all models, see Table S1 in supplementary material). The best model(s) for each life stage was/were selected based on their simplicity using their AICc value, ΔAICc and Akaike weight (Burnham and Anderson, 2002). In all analyses, population and family levels were initially treated as random effects nested within the broader levels of ancestry and, ancestry and population, respectively. Following our initial model selection, AICc analysis was repeated, but this time excluding family as a random effect. We reasoned that family would show considerable variation due to maternal effects, and would mask other potentially important factors (e.g. Cook *et al.*, 2018). In our AICc tables, we therefore report models with and without family included as a random effect. We then ran traditional statistical analyses (e.g. GLM) on the top models (s), followed by *post-hoc* Tukey’s HSD tests to compare among treatments. All statistical analyses were conducted in JMP 11 (SAS Institute Inc., Cary, NC). Note that some data loss occurred (~40 trials from the cold–cold treatment) due to computer breakdown, and therefore not all sample sizes are equivalent. When body mass appeared in the top model for RMR, the metabolic rate was reported as ‘mass-adjusted’ rather than ‘mass-specific.’ This was because the effect of body mass was accounted for statistically, rather than by dividing the RMR by the mass of each individual (following Cook *et al.*, 2018).

## Results

**Figure 2:**
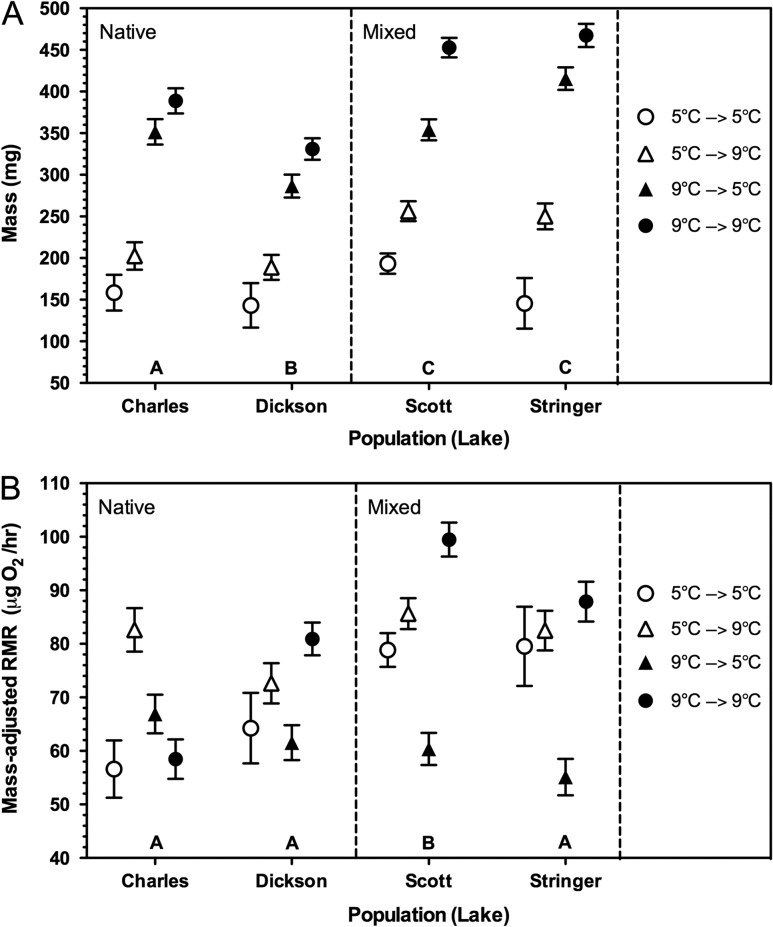
Differences in (**A**) mass and (**B**) mass-adjusted routine metabolic rate of single brook trout fry, initially reared at 5°C or 9°C as eggs and alevin. Fry were then exposed to either matched (circles) or mismatched (triangles) temperatures for 30 days and measured. Although populations originated from either native or hatchery-introgressed ancestry, ancestry was not in the top AICc model. Letters above population names indicate significant differences between populations (Tukey’s HSD, *P* < 0.05). In panel (A), initial rearing and final acclimation temperatures were both in the top AICc model; significant differences (*P* < 0.05) among treatments are detailed in the text. The interaction between the two temperature terms was present in the second strongly supportive AICc model but was not statistically significant. In panel (B), initial rearing temperature was not in the top AICc model, but final acclimation temperature was, with fry acclimated to 9°C having higher mass-adjusted RMR than those at 5°C (*P* < 0.05). There was a significant interaction between population and final acclimation temperature (*P* < 0.05), indicating that populations responded differently to the final acclimation temperature (detailed in text). Panels (A) and (B) both show LSM ± SEM from general linear mixed-models, with fry mass included as a covariate in panel (B).

Mass of fry was influenced by a single strongly supported AICc model including population, family, initial rearing temperature, final acclimation temperature, and the interaction of the two temperatures (Model 1, Table 2). A second model with moderate support (ΔAICc between 2 and 6) had similar terms as Model 1, but did not include the interaction term (Model 2, Table 2). Model 1 was 77% likely to be the best explanatory model compared with only 23% for Model 2 (Wi, Table 2). Additionally, the evidence ratio (ER) associated with Model 2 of 3.34 in Table 2 indicated that Model 1 was at least three times more likely to be the best model than Model 2. When the random effect of family was excluded from the analysis, two models strongly supported the data (models i and ii, ΔAICc < 2, Table 2). Both models included population of origin, initial rearing temperature and final acclimation temperature, with Model ii also including the interaction between the two temperatures. Because Model i had the strongest support, was the simplest model, and the interaction term in Model ii was not significant (*P* = 0.89), we focussed subsequent analyses on Model i. All three terms in Model i were significant in contributing to variation in the mass of fry (population: *F*_3,478_ = 36.88, *P* < 0.0001; initial temperature *F*_1,478_ = 581.24, *P* < 0.0001; final temperature *F*_1,478_ = 64.25, *P* < 0.0001). Fry from Scott Lake and Stringer Lake had similar mass, but were significantly heavier than fry from Charles Lake whose fry were also significantly larger than those from Dickson (Fig. 2A). When accounting for population and final acclimation temperature, individuals initially reared at 9°C were heavier than their siblings initially reared at 5°C (LSM ± SE; 9°C: 380.24 ± 4.87 mg and 5°C: 195.49 ± 6.06 mg; solid symbols versus open symbols, Fig. 2A). This was also true for the final acclimation temperature, controlling for population and initial rearing temperature; fry tested at 9°C were heavier than their siblings tested in colder waters of 5°C (LSM ± SE; 9°C: 318.58 ± 5.01 mg and 5°C: 257.15 ± 5.87 mg). A lack of interaction between the initial rearing temperature and the final acclimation temperature on the body mass of fry indicated that the temperature experienced at one life stage did not impact how that individual would respond to temperature at a subsequent life stage.

**Table 2:** Summary of Akaike’s Information Criteria (AIC) models predicting variability in mass and routine metabolic rate (RMR) of brook trout fry

	Models	K	AICc	ΔAICc	ER	Wi
Mass	**1. Pop + Fam[Pop]&Random + Temp**_**Init**_**+ Temp**_**Final**_**+ Temp**_**Init**_**× Temp**_**Final**_	**7**	**5595.33**	**0.00**	**1.00**	**0.77**
	2. Pop + Fam[Pop]&Random + Temp_Init_ + Temp_Final_	6	5597.74	2.41	3.34	0.23
	**i. Pop + Temp**_**Init**_**+ Temp**_**Final**_	**5**	**5630.77**	**0.00**	**1.00**	**0.53**
	**ii. Pop + Temp**_**Init**_**+ Temp**_**Final**_**+ Temp**_**Init**_**× Temp**_**Final**_	**6**	**5631.05**	**0.28**	**1.15**	**0.46**
RMR	**A. Pop + Fam[Pop]&Random + Mass + Temp**_**Final**_**+ Pop × Temp**_**Final**_	**7**	**4282.42**	**0.00**	**1.00**	**0.89**
	B. Pop + Fam[Pop]&Random + Mass + Temp_Init_ + Temp_Final_ + Temp_Init_ × Temp_Final_	8	4286.82	4.40	9.03	0.10
	**a. Pop + Mass + Temp**_**Final**_**+ Pop × Temp**_**Final**_	**6**	**4293.04**	**0.00**	**1.00**	**0.76**
	b. Pop + Mass + Temp_Init_ + Temp_Final_ + Temp_Init_ × Temp_Final_	7	4295.50	2.46	3.42	0.22

Models numbered with numeric or capital letters include family and those labelled with roman numerals and lower case letters exclude family from the analysis. Bolded models have strong support for predicting variability (**Δ**AICc between 0 and 2) and models in regular font have moderate support (**Δ**AICc between 2 and 6).

Note: Candidate models were ordered by ascending AICc value. The number identifying each model corresponds with that model’s location within 25 or 17 candidate models for mass and 54 or 39 for RMR. RMR = routine metabolic rate; K = number of parameters in the model plus two (for the intercept and variance); AICc = Akaike’s Information Criteria corrected for small sample sizes; ΔAICc = difference in AICc score between focal model and best model; ER (evidence ratio) = measure of how much more likely the best model is than the focal model; W_i_ (Akaike’s weight) = probability that focal model is the best approximating model; An = ancestry; Pop = population; Fam = family; Mass = mass of single organism; Temp_Init_ = initial rearing temperature from eggs to yolk absorption; Temp_Final_ = final acclimation temperature experienced by fry at which measurements were taken.

Variation in the RMR of brook trout fry was accounted for by a single strongly supported model (Model A) including population, family, mass, final acclimation temperature, and the interaction between population and final temperature (Table 2), indicating that the RMR of populations responded differently to the final acclimation temperature. A second model with moderate support (Model B) included population, family, mass, initial rearing temperature, final temperature and the interaction of the two temperatures (instead of the population by final temperature interaction). However, Model B was nine times less likely to be the best model (ER = 9.03) than Model A and had only a 10% chance of being the top model compared with 89% for Model A (Wi, Table 2). The same top two models as above occurred when the random effect of family was removed from the AICc, except that they did not include family (models a and b, Table 2). Model a was strongly supportive (ΔAICc < 2) with a 76% likelihood of being the best approximating model, over a 22% chance for the moderately supportive Model b (Wi, ΔAICc between 2 and 6). Refuting our hypothesis, ancestry did not impact metabolic differences throughout development, because it never appeared in any strongly or moderately supporting model. All parameters in Model a were significant in accounting for variability in RMR of fry (population: *F*_3,475_ = 14.01, *P* < 0.0001; mass: *F*_1,475_ = 301.61, *P* < 0.0001; final temperature: *F*_1,475_ = 85.37, *P* < 0.0001; population × final temperature: *F*_3,475_ = 5.11, *P* = 0.0017). On average, fry from Charles, Dickson and Stringer lakes had significantly lower mass-adjusted RMRs than fry from Scott Lake when accounting for temperature differences (*P* < 0.05, Fig. 2B). When fry were acclimated to 9°C (closed circles or open triangles), no matter what temperature they were initially reared at as eggs/alevin, they had higher mass-adjusted RMRs than their siblings acclimated to 5°C (open circles or closed triangles). The exception to this was Charles Lake, in which fry reared entirely at 9°C (closed circles) had some of the lowest mass-adjusted RMRs, while fry from the other populations in this temperature treatment had among the highest mass-adjusted RMR (Fig. 2B).

## Discussion

The mass of brook trout fry was influenced by their population of origin, as well as initial rearing and final acclimation temperatures experienced throughout development. RMR was strongly influenced by population, mass and final acclimation temperature, with individuals experiencing warmer waters at the time of testing having higher mass-adjusted RMR than individuals acclimated to colder waters. Interestingly, mass-adjusted RMR varied among the four populations, depending on the final temperature experienced at the time of testing. Although we hypothesized that native vs introgressed ancestry would be an important contributing factor in how fry would respond to changes in temperature, this was not the case, as this level of ancestry never appeared as a significant variable in any of the top AICc models.

A substantial amount of variation in fry body mass and mass-adjusted metabolic rate was accounted for by family effects, consistent with findings for earlier life stages (Cook *et al.*, 2018). Although we cannot partition the relative contribution of genetic variation and maternal resource allocation to phenotypic variation in fry, we suspect that maternal effects likely played a prominent role. In fish, egg size is a direct indicator of maternal investment, and many studies have found egg size to directly affect juvenile size and growth rate, which in turn can affect survival (Einum and Fleming, 1999; Marshall and Keough, 2007; Venturelli *et al.*, 2010). In addition to maternal effects contributing to intraspecific variation in offspring mass, heritable genetic differences in growth rate from eggs to juveniles have also been reported in other fish species (García-Celdrán *et al.*, 2015; Janhunen *et al.*, 2016; Leeds *et al.*, 2016). Identifying the extent to which fry body mass was determined by maternal provisioning or heritable genetic differences within and among populations was, however, beyond the scope of this study.

Mass-adjusted RMR of fry also varied greatly among families. As was the case with mass, some of this physiological variation was likely due to environmental and/or maternal effects (reviewed in, Burton *et al.*, 2011; Metcalfe *et al.*, 2016). For example, RMR of Atlantic salmon alevin decreases with increasing egg volume (Rossignol *et al.*, 2010), while the standard metabolic rate of brown trout offspring can vary depending on the position an individual occupied within the egg mass (Burton *et al.*, 2013). The mechanistic basis for such differences among individuals remains unknown, although metabolic rate can be modulated by maternal hormones, including testosterone and glucocorticoids (Tobler *et al.*, 2007; Sloman, 2010).

Across taxa, numerous studies have also shown that RMR has a genetic component (e.g. birds (*Saxicola torquata*; Tieleman *et al.*, 2009); mammals (*Myodes glareolus*; Boratyński *et al.*, 2013); fish (*Acanthochromis polyacanthus*; Munday *et al.*, 2017)). Family level-variation in RMR and the performance consequences of this variation have been reported previously, at least with respect to egg metabolism (Robertsen *et al.*, 2014). For example, offspring of Atlantic salmon (*Salmo salar*) families that differed in egg metabolic rates differed in performance, depending on the tributary in which they developed (Robertsen *et al.*, 2014). Variation in RMR has also been linked with a variety of organismal traits, including aggression and dominance (reviewed in, Metcalfe *et al.*, 2016), and propensity for movement (Myles-Gonzalez *et al.*, 2015). We did not study behavioural traits or movement ecology, but the existence of family-level variation in RMR of fry suggests that families may also differ in behaviour or performance (e.g. Robertsen *et al.*, 2014; Metcalfe *et al.*, 2016).

We detected significant differences among populations in the mass of fry. Such phenotypic variation has been widely reported in juvenile salmonids, with contributing factors including maternal investment, egg incubation temperature and whether individuals were wild or captive-bred (Braun *et al.*, 2013; Siikavuopio *et al.*, 2013). The apparent lack of effect of ancestry suggests that carry-over effects from hatchery conditions likely played a minimal role. Population-variation in egg size has been widely reported in salmonids (Fleming and Gross, 1990), and egg size predicts juvenile mass (e.g. Braun *et al.*, 2013). Unfortunately, because we did not measure egg size we cannot comment on the extent to which it may have varied among populations.

The observed differences in mass-adjusted fry RMR among our study populations were consistent with reported intraspecific differences in juvenile metabolic phenotypes for other salmonids, with differences consistent with local adaptation (Lahti *et al.*, 2002; Seppänen *et al.*, 2009; Stitt *et al.*, 2014). For example, when brook trout (Stitt *et al.*, 2014) and Atlantic salmon (Seppänen *et al.*, 2009) were reared under shared hatchery conditions at relatively low temperatures, populations from more northern latitudes had lower metabolic rates than populations from lower latitudes. The optimal metabolic phenotype presumably varies, however, with environmental context (Burton *et al.*, 2011). High RMRs may confer a selective advantage only under conditions such as high environmental productivity, low habitat complexity, and/or predictable food supplies (Lahti *et al.*, 2002; Reid *et al.*, 2012). In our study, the specific environmental factors contributing to population-level differences in RMR, and the relative fitness costs and benefits of differing metabolic phenotypes, currently remain unknown.

In fish, temperature is a critical determinant of time to hatch, growth rate and mass (Marten, 1992; Başçinar and Okumuş, 2002; Jonsson and Jonsson, 2014). Previously, we showed that higher temperature increased rates of egg development, but had a negative effect on the size of resultant brook trout alevin (Cook *et al.*, 2018). Similar results have been reported in brook trout (Marten, 1992; Başçinar and Okumuş, 2002) and brown trout (Réalis-Doyelle *et al.*, 2016). In this study, we showed that both the initial rearing temperature (of eggs and alevin combined) and final acclimation temperatures (of fry) independently had positive effects on growth of brook trout fry, as mass increased with increasing temperature. Typically, growth rate increases with increasing temperature up to an optimum, beyond which growth rate drastically declines (Jonsson and Jonsson, 2014). Presumably, we did not detect a negative impact of warmer temperature on the size of fry, as 9°C was not beyond the thermal optimum for the fry life stage (Biro, 1998).

Environmental temperature has been shown to influence metabolic processes across multiple life stages. For example, embryonic temperature has lasting impacts on swim performance and metabolic enzyme activity in zebrafish (Scott and Johnston, 2012; Schnurr *et al.*, 2014). In three-spined stickleback (*Gasterosteus aculeatus*), low or high embryonic temperatures have negative effects on growth rate, which are manifested as a reproductive cost (Lee *et al.*, 2013). Although we found that acclimation temperature of fry was a significant predictor of mass-adjusted RMR, embryonic temperature had no apparent effect. We are not aware of any study of fish showing carry-over effects of embryonic temperature on whole animal mass-adjusted RMR at later life stages, although such patterns have been detected in other taxa (e.g. birds, Ben-Ezra and Burness, 2017). Not surprisingly, we found that acclimation temperature had a positive effect on mass-adjusted RMR of fry, although not equally across populations (see below). Positive relations between mass-adjusted RMR and temperature have been widely reported in warm (e.g. Indian major carp (*Labeo rohita*, *Catla catla*, *Cirrhinus mrigala*), Das *et al.*, 2004) and cold-adapted species (e.g. brook trout, Stitt *et al.*, 2014; Antarctic fish (*Lepidonotothen squamifrons*, *L. nudifrons*, *Trematomus hansoni*), Sandersfeld *et al.*, 2017).

We detected a significant statistical interaction between population and acclimation temperature, with fry from Charles Lake displaying lower mass-adjusted RMRs when acclimated to warm temperatures than fry from other populations. Differences among populations in their capacity for temperature acclimation have been reported for cold- and warmwater species (e.g. juvenile brook trout, Stitt *et al.*, 2014; adult coral reef damselfish, *A. polyacanthus*, Donelson and Munday, 2012). However, given that the four populations in our study were from similar latitudes, and presumably exposed to similar temperature regimes over time, differences among populations were unexpected. Currently, we cannot say whether the observed differences in mass-adjusted RMR reflect adaptation to differing thermal regimes (e.g. Stitt *et al.*, 2014) or other environmental factors (e.g. food availability and its predictability, Reid *et al.*, 2012).

Environments individuals experience at one life stage have been hypothesized to mediate the responses of individuals at subsequent life stages (e.g. Monaghan, 2008). Ectotherms are strongly influenced by temperature, and thus it is possible that once an individual has been primed to a specific temperature, the capacity for subsequent morphological and physiological deviations is limited. For example, warmwater zebrafish embryos reared at one temperature, and then tested at the same temperature as adults, had higher swimming speeds than individuals that experienced a mismatched rearing and testing environment (Scott and Johnston, 2012). This was likely due to the differences in the composition of muscle fibre types between individuals at the different acclimation temperatures (Scott and Johnston, 2012).

Using a coldwater salmonid, we sought evidence for an effect of environmental matching/mismatching across life stages on the morphological and metabolic phenotype of fry. Evidence for mismatching would exist if the Temp_init_ × Temp_final_ interaction term was retained in a strongly supported statistical model (i.e. one with ΔAICc < 2). For fry mass, a Temp_init_ × Temp_final_ interaction term did appear in one of the top models, both with and without the inclusion of family identity (Models 1 and ii, respectively, Table 2). However, because there existed a simpler model, with a similar level of support (i.e. Model i), we chose to focus on this simpler model. Nonetheless, there appears to be some evidence that the growth of fry at a given temperature was dependent on the temperature experienced at the egg/alevin life stage. For example, fry acclimated to 9°C that had also been exposed to 9°C as eggs and alevin, were larger than their siblings initially reared as eggs and alevin at 5°C. However, in this analysis it is difficult to disentangle the effect of environmental matching from a simple Q10 effect.

In the case of mass-adjusted RMR, there was little evidence for carry-over effects between life stages. The initial temperature individuals experienced as eggs and alevin did not predict the metabolic rate of fry; fry were largely dependent on the final temperature they experienced at testing. This contrasts with a recent study on another coldwater salmonid, lake whitefish (Mueller *et al.*, 2015). In that species, the costs of development (i.e. the amount of oxygen necessary to produce a milligram of hatchling mass) differed depending on whether temperatures were constant or changed (increased or decreased) throughout egg development. Generally, costs of development became higher as temperatures increased above the predicted thermal optimum, but costs were reduced if individuals had been previously incubated at colder temperatures (Mueller *et al.*, 2015). Given the life-history of brook trout in which eggs are incubated at cold temperatures followed by individuals later moving into warmer waters (Power, 1980; Biro, 1998) we might have expected a similar result. However, a statistical interaction between initial and final temperature only appeared in models with moderate support (ΔAICc between 2 and 6); the interaction did not appear in strongly supported models. Why prior incubation temperature appears to produce contrasting effects on energy metabolism in developing lake whitefish and brook trout is not clear.

### Conservation implications

Although family- and population-level variation in mass and mass-adjusted RMR suggests these phenotypic traits likely have a heritable component, our study design was unable to distinguish genetic versus maternal effects. As populations differed in their metabolic response to acclimation temperature, some populations may cope better than others if faced with synergistic stressors. For example, a low mass-adjusted RMR may be favourable if faced with decreased food availability (e.g. Burton *et al.*, 2011), and thus promote survival if individuals are also experiencing warming temperatures. Currently, we know little about the thermal tolerance of various populations of brook trout at these early developmental stages, which are presumed to be the most vulnerable to environmental variation. Future studies should focus on how thermal tolerance changes among life stages, and whether there is the capacity for experiences at one life stage to influence the thermal tolerance at subsequent life stages, as well as resolving trait heritability.

As environmental temperatures continue to increase, populations of coldwater fish species are likely to come under increasing physiological stress (Whitney *et al.*, 2016). Brook trout are particularly vulnerable to the effects of climatic change (Meisner, 1990; Flebbe *et al.*, 2006), and are predicted to experience a dramatic (49%) decrease across their range from direct and indirect climate change effects (Chu *et al.*, 2005; Flebbe *et al.*, 2006). Although assisted migration could potentially increase the adaptive capacity of populations of brook trout and other stenothermal species to climate change-related stressors (Aitken and Whitlock, 2013; Carlson *et al.*, 2014), the results of this study failed to show significant differences or benefits in thermal performance for introgressed versus native populations at the life stages we examined. As assisted gene flow between populations with differing adaptations also includes some inherent risks, potential efforts should be evaluated thoroughly before adopting evolutionary rescue as a management tool (Garant *et al.*, 2007; Aitken and Whitlock, 2013). Future studies investigating the heritability versus plasticity for ecologically important physiological traits (Hutchings, 2011), particularly in wild populations (Charmantier and Garant, 2005; Dufresne *et al.*, 2015), would be extremely useful for informing management and conservation efforts of coldwater populations and species facing rapidly changing environmental conditions.

## Supplementary Material

Supplementary Data
