# Supplementary material for: Impacts of environmental matching on the routine metabolic rate and mass of native and mixed-ancestry brook trout (Salvelinus fontinalis) fry
Source: Conserv Physiol. 2018 May 8;6(1):coy023. doi: 10.1093/conphys/coy023 (PMC6194207; doi:10.1093/conphys/coy023)
Supplement: Supplementary Data [file coy023cook_conphys-2017-030_r2_supplmaterial.docx]

## Supplementary material


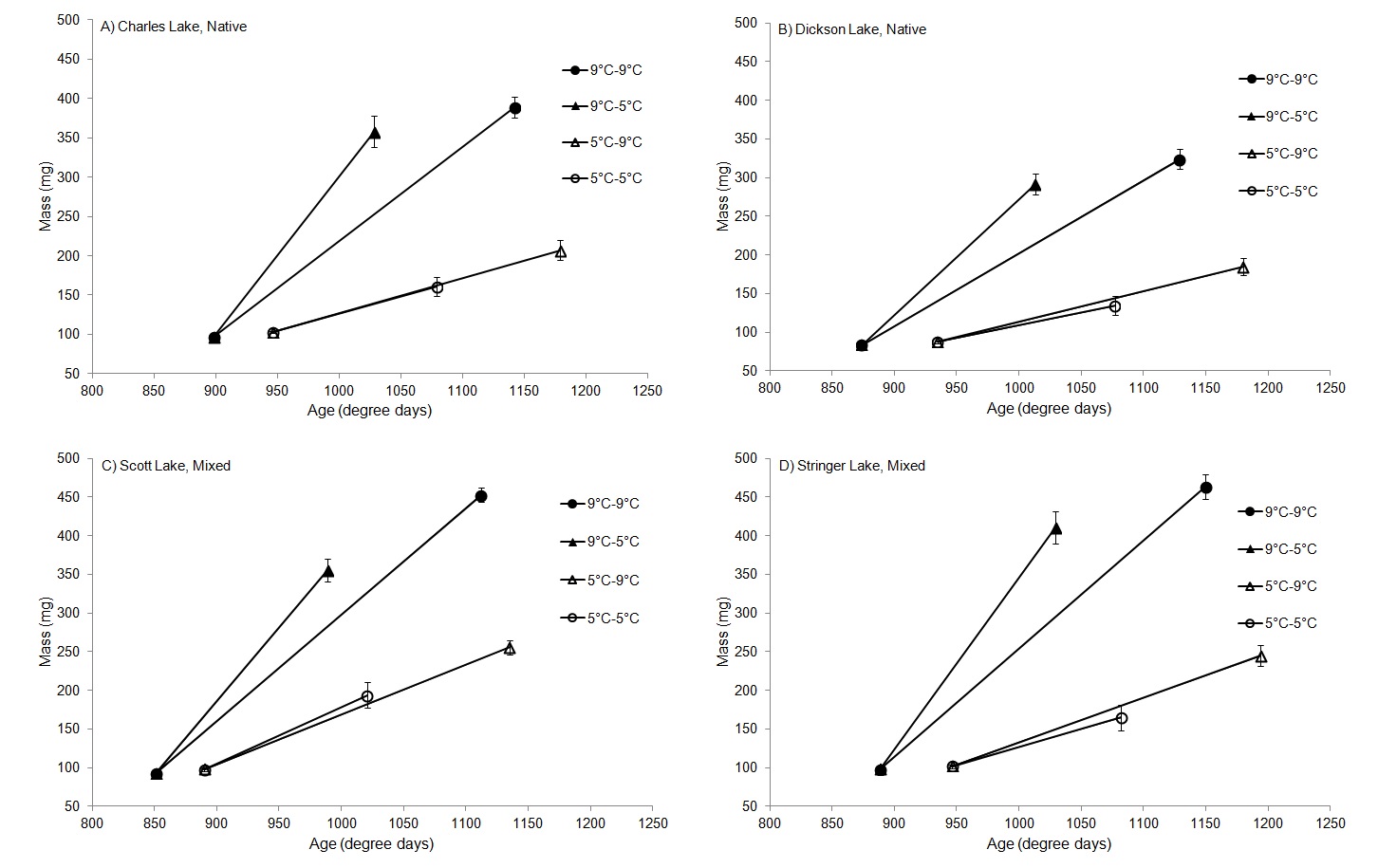


**Supplementary Figure S1**. Growth rates of populations of brook trout fry (mean and standard error) bred from wild parents with native or introgressed ancestry. Initial mass data are for alevin at yolk absorption (Cook et al. 2018). On average, alevin at yolk absorption were ca. 5% heavier when incubated at 5°C than 9°C. Individuals were then acclimated to either 5°C or 9°C for 30 calendar days and weighed; A) Charles Lake, Native, B) Dickson Lake, Native, C) Scott Lake, Mixed, and D) Stringer Lake, Mixed.

**Supplementary Table S1**: Summary of all Akaike Information Criteria (AIC) models showing their relative fit for describing observed variation in mass and routine metabolic rate (RMR) of brook trout fry. Models and outcome parameters in bold show strongly supported models (delta AICc between 0 & 1.99), models shown in italics had moderate support (delta AICc between 2 and 6), and models shown in regular font were not supported (delta AICc greater than 6). The parameters included in the AICc models were ancestry (native vs. hatchery-introgressed), population (Charles, Dickson, Scott and Stringer Lakes), family identity (one of six families per population, except for Charles Lake (four families only)), and two temperature terms (initial rearing temperature from eggs to yolk absorption, R.Temp; and final acclimation temperature experienced by fry at which measurements were performed, F. Temp). Population and family levels were treated as random effects and nested within the larger level(s) of ancestry, and ancestry/population, respectively when those larger levels occurred in the model. Candidate models are ordered in ascending AICc values.

**(A) Mass of fry (family and population nested within ancestry)**

| **Model** | **AICc** | **delta(i)= di** | **exp(-0.5*di)** | **ER** | **wi** | **Additive wi** |
| --- | --- | --- | --- | --- | --- | --- |
|  |  |  |  |  |  |  |
| **Pop+Fam[Pop]&Random+ R.Temp+F.Temp+ R.Temp:F.Temp** | **5595.33** | **0.00** | **1** | **1** | **0.767031448** | **0.767031448** |
| *Pop+Fam[Pop]&Random+R.Temp+ F.Temp* | *5597.74* | *2.41* | *0.299692* | *3.336759078* | *0.229873188* | *0.996904636* |
| An+Pop[An]&Random+Fam[An,Pop]&Random+R.Temp+F.Temp+ R.Temp:F.Temp | 5606.50 | 11.17 | 0.00375375 | 266.4002827 | 0.002879244 | 0.99978388 |
| An+Pop[An]&Random+Fam[An,Pop]&Random+R.Temp+F.Temp | 5611.68 | 16.35 | 0.000281606 | 3551.054924 | 0.000216001 | 0.999999881 |
| Fam+R.Temp+F.Temp | 5627.25 | 31.92 | 1.17128E-07 | 8537681.143 | 8.98407E-08 | 0.999999971 |
| Fam+R.Temp+F.Temp+ R.Temp:F.Temp | 5629.50 | 34.17 | 3.80258E-08 | 26297909.31 | 2.9167E-08 | 1 |
| Pop+Fam[Pop]&Random+ R.Temp+Pop:R.Temp | 5644.48 | 49.15 | 2.12429E-11 | 47074627568 | 1.62939E-11 | 1 |
| Pop+Fam[Pop]&Random+R.Temp | 5664.65 | 69.32 | 8.85836E-16 | 1.12888E+15 | 6.79464E-16 | 1 |
| An+Pop[An]&Random+Fam[An,Pop]&Random+R.Temp+An:R.Temp | 5671.77 | 76.44 | 2.51921E-17 | 3.96949E+16 | 1.93232E-17 | 1 |
| An+Pop[An]&Random+Fam[An,Pop]&Random+R.Temp | 5679.91 | 84.58 | 4.30216E-19 | 2.32441E+18 | 3.29989E-19 | 1 |
| Fam+R.Temp | 5694.16 | 98.83 | 3.46209E-22 | 2.88843E+21 | 2.65553E-22 | 1 |
| Fam+R.Temp+Fam:R.Temp | 5717.85 | 122.52 | 2.48382E-27 | 4.02606E+26 | 1.90517E-27 | 1 |
| R.Temp+F.Temp | 5735.84 | 140.51 | 3.08064E-31 | 3.24608E+30 | 2.36295E-31 | 1 |
| R.Temp+F.Temp+R.Temp:F.Temp | 5737.74 | 142.41 | 1.19141E-31 | 8.39341E+30 | 9.13849E-32 | 1 |
| R.Temp | 5778.35 | 183.02 | 1.81014E-40 | 5.52444E+39 | 1.38843E-40 | 1 |
| Pop+Fam[Pop]&Random+F.Temp+ Pop:F.Temp | 5972.97 | 377.64 | 9.9201E-83 | 1.00805E+82 | 7.60903E-83 | 1 |
| Pop+Fam[Pop]&Random+F.Temp | 5996.91 | 401.58 | 6.28074E-88 | 1.59217E+87 | 4.81753E-88 | 1 |
| An+Pop[An]&Random+Fam[An,Pop]&Random+F.Temp+An:F.Temp | 6005.01 | 409.68 | 1.09425E-89 | 9.13864E+88 | 8.39328E-90 | 1 |
| Pop+Fam[Pop]&Random | 6011.66 | 416.33 | 3.93631E-91 | 2.54045E+90 | 3.01927E-91 | 1 |
| An+Pop[An]&Random+Fam[An,Pop]&Random+F.Temp | 6012.90 | 417.57 | 2.11752E-91 | 4.72252E+90 | 1.6242E-91 | 1 |
| An+Pop[An]&Random+Fam[An,Pop]&Random | 6029.11 | 433.78 | 6.39543E-95 | 1.56362E+94 | 4.9055E-95 | 1 |
| Fam+F.Temp | 6043.99 | 448.66 | 3.75594E-98 | 2.66245E+97 | 2.88093E-98 | 1 |
| Fam | 6054.03 | 458.70 | 2.4806E-100 | 4.0312E+99 | 1.9027E-100 | 1 |
| F.Temp | 6059.41 | 464.08 | 1.6839E-101 | 5.9387E+100 | 1.2916E-101 | 1 |
| Fam+F.Temp+Fam:F.Temp | 6069.01 | 473.68 | 1.3858E-103 | 7.2161E+102 | 1.0629E-103 | 1 |
|  |  | Sum: | 1.303727511 |  |  |  |

**(B) Mass of fry (population nested within ancestry; family not included as a predictive variable)**

| **Model** | **AICc** | **delta(i)= di** | **exp(-0.5*di)** | **ER** | **wi** | **Additive wi** |
| --- | --- | --- | --- | --- | --- | --- |
|  |  |  |  |  |  |  |
| **Pop+R.Temp+F.Temp** | **5630.77** | **0.00** | **1** | **1** | **0.532872077** | **0.532872077** |
| **Pop+R.Temp+F.Temp+ R.Temp:F.Temp** | **5631.05** | **0.28** | **0.869358235** | **1.150273799** | **0.463256729** | **0.996128806** |
| An+Pop[An]&Random+R.Temp+ F.Temp | 5641.23 | 10.46 | 0.005353525 | 186.7928035 | 0.002852744 | 0.99898155 |
| An+Pop[An]&Random+R.Temp+ F.Temp+R.Temp:F.Temp | 5643.29 | 12.52 | 0.001911246 | 523.2189401 | 0.00101845 | 1 |
| An+Pop[An]&Random+R.Temp+ An:R.Temp | 5686.15 | 55.38 | 9.42727E-13 | 1.06075E+12 | 5.02353E-13 | 1 |
| An+Pop[An]&Random+R.Temp | 5692.14 | 61.37 | 4.71709E-14 | 2.11995E+13 | 2.5136E-14 | 1 |
| Pop+R.Temp+Pop:R.Temp | 5695.59 | 64.82 | 8.40458E-15 | 1.18983E+14 | 4.47856E-15 | 1 |
| Pop+R.Temp | 5700.21 | 69.44 | 8.34249E-16 | 1.19868E+15 | 4.44548E-16 | 1 |
| R.Temp+F.Temp+R.Temp:F.Temp | 5737.74 | 106.97 | 5.91234E-24 | 1.69138E+23 | 3.15052E-24 | 1 |
| R.Temp | 5778.35 | 147.58 | 8.98277E-33 | 1.11324E+32 | 4.78667E-33 | 1 |
| An+Pop[An]&Random+F.Temp+ An:F.Temp | 6008.76 | 377.99 | 8.3275E-83 | 1.20084E+82 | 4.43749E-83 | 1 |
| An+Pop[An]&Random+F.Temp | 6013.88 | 383.11 | 6.43755E-84 | 1.55339E+83 | 3.43039E-84 | 1 |
| Pop+F.Temp+Pop:F.Temp | 6019.02 | 388.25 | 4.92701E-85 | 2.02963E+84 | 2.62547E-85 | 1 |
| Pop+F.Temp | 6024.29 | 393.52 | 3.5336E-86 | 2.82997E+85 | 1.88296E-86 | 1 |
| An+Pop[An]&Random | 6027.28 | 396.51 | 7.92406E-87 | 1.26198E+86 | 4.22251E-87 | 1 |
| Pop | 6033.83 | 403.06 | 2.99663E-88 | 3.33708E+87 | 1.59682E-88 | 1 |
| F.Temp | 6059.41 | 428.64 | 8.35616E-94 | 1.19672E+93 | 4.45276E-94 | 1 |
|  |  | Sum: | 1.876623006 |  |  |  |

**(C) RMR of fry (family and population nested within ancestry)**

| **Model** | **AICc** | **delta(i)= di** | **exp(-0.5*di)** | **ER** | **wi** | **Additive wi** |
| --- | --- | --- | --- | --- | --- | --- |
|  |  |  |  |  |  |  |
| **Pop+Fam[Pop]&Random+Mass+F.Temp+Pop:F.Temp** | **4282.42** | **0.00** | **1** | **1** | **0.885548389** | **0.885548389** |
| *Pop+Fam[Pop]&Random+Mass+R.Temp+F.Temp+R.Temp:F.Temp* | *4286.82* | *4.40* | *0.110803158* | *9.025013499* | *0.098121558* | *0.983669947* |
| An+Pop[An]&Random+Fam[An,Pop]&Random+Mass+R.Temp+F.Temp+R.Temp:F.Temp | 4290.48 | 8.06 | 0.01777433 | 56.26091125 | 0.015740029 | 0.999409976 |
| Pop+Fam[Pop]&Random+Mass+ R.Temp+F.Temp | 4297.90 | 15.48 | 0.000435072 | 2298.472383 | 0.000385277 | 0.999795253 |
| Pop+Fam[Pop]&Random+Mass+F.Temp | 4299.33 | 16.91 | 0.000212834 | 4698.506635 | 0.000188474 | 0.999983728 |
| An+Pop[An]&Random+Fam[An,Pop]&Random+Mass+R.Temp+F.Temp | 4304.75 | 22.33 | 1.41613E-05 | 70615.15073 | 1.25405E-05 | 0.999996268 |
| An+Pop[An]&Random+Fam[An,Pop]&Random+Mass+F.Temp | 4307.52 | 25.10 | 3.5449E-06 | 282095.2334 | 3.13918E-06 | 0.999999407 |
| Fam+Mass+R.Temp+F.Temp+R.Temp:F.Temp | 4310.90 | 28.48 | 6.54104E-07 | 1528809.674 | 5.7924E-07 | 0.999999987 |
| Fam+Mass+F.Temp | 4320.36 | 37.94 | 5.77343E-09 | 173207351.8 | 5.11265E-09 | 0.999999992 |
| Fam+Mass+R.Temp+F.Temp | 4321.15 | 38.73 | 3.88944E-09 | 257106254.7 | 3.44429E-09 | 0.999999995 |
| Mass+R.Temp+Mass:F.Temp | 4321.51 | 39.09 | 3.24874E-09 | 307812072.3 | 2.87691E-09 | 0.999999998 |
| Mass+R.Temp+F.Temp+ R.Temp:F.Temp | 4322.27 | 39.85 | 2.22168E-09 | 450108849.8 | 1.96741E-09 | 1 |
| Mass+R.Temp+F.Temp | 4334.56 | 52.14 | 4.76368E-12 | 2.09922E+11 | 4.21847E-12 | 1 |
| Fam+Mass+F.Temp+Fam:F.Temp | 4335.52 | 53.10 | 2.94769E-12 | 3.39249E+11 | 2.61032E-12 | 1 |
| Mass+F.Temp | 4339.00 | 56.58 | 5.17379E-13 | 1.93282E+12 | 4.58164E-13 | 1 |
| Pop+Fam[Pop]&Random+Mass+ R.Temp+Pop:R.Temp | 4362.26 | 79.84 | 4.60219E-18 | 2.17288E+17 | 4.07546E-18 | 1 |
| Pop+Fam[Pop]&Random+Mass+ R.Temp | 4373.40 | 90.98 | 1.75365E-20 | 5.70238E+19 | 1.55295E-20 | 1 |
| An+Pop[An]&Random+Fam[An,Pop]&Random+Mass+R.Temp | 4381.98 | 99.56 | 2.40337E-22 | 4.16082E+21 | 2.1283E-22 | 1 |
| Pop+Fam[Pop]&Random+Mass | 4389.08 | 106.66 | 6.90359E-24 | 1.44852E+23 | 6.11347E-24 | 1 |
| Mass+R.Temp | 4393.71 | 111.29 | 6.81842E-25 | 1.46662E+24 | 6.03804E-25 | 1 |
| Mass+R.Temp+Mass:R.Temp | 4394.24 | 111.82 | 5.23113E-25 | 1.91163E+24 | 4.63242E-25 | 1 |
| Fam+Mass+R.Temp | 4395.67 | 113.25 | 2.55903E-25 | 3.90773E+24 | 2.26614E-25 | 1 |
| An+Pop[An]&Random+Fam[An,Pop]&Random+Mass | 4398.69 | 116.27 | 5.65315E-26 | 1.76893E+25 | 5.00614E-26 | 1 |
| Pop+Fam[Pop]&Random+Mass+ Pop:Mass | 4401.06 | 118.64 | 1.72843E-26 | 5.7856E+25 | 1.53061E-26 | 1 |
| An+Pop[An]&Random+Fam[An,Pop]&Random+Mass+An:Mass | 4408.19 | 125.77 | 4.89093E-28 | 2.0446E+27 | 4.33116E-28 | 1 |
| Fam+Mass | 4412.24 | 129.82 | 6.45573E-29 | 1.54901E+28 | 5.71686E-29 | 1 |
| Mass | 4413.65 | 131.23 | 3.18983E-29 | 3.13496E+28 | 2.82475E-29 | 1 |
| Pop+Fam[Pop]&Random+R.Temp+ F.Temp+ R.Temp:F.Temp | 4415.21 | 132.79 | 1.46224E-29 | 6.83884E+28 | 1.29488E-29 | 1 |
| An+Pop[An]&Random+Fam[An,Pop]&Random+R.Temp+F.Temp+ R.Temp:F.Temp | 4417.27 | 134.85 | 5.22029E-30 | 1.9156E+29 | 4.62282E-30 | 1 |
| Fam+Mass+R.Temp+Fam:R.Temp | 4421.48 | 139.06 | 6.3607E-31 | 1.57215E+30 | 5.6327E-31 | 1 |
| Pop+Fam[Pop]&Random+R.Temp+ F.Temp | 4423.77 | 141.35 | 2.02413E-31 | 4.9404E+30 | 1.79246E-31 | 1 |
| An+Pop[An]&Random+Fam[An,Pop]&Random+R.Temp+F.Temp | 4429.31 | 146.89 | 1.26836E-32 | 7.88421E+31 | 1.12319E-32 | 1 |
| Fam+Mass+Fam:Mass | 4435.68 | 153.26 | 5.24824E-34 | 1.9054E+33 | 4.64757E-34 | 1 |
| Fam+R.Temp+F.Temp+ R.Temp:F.Temp | 4449.06 | 166.64 | 6.52505E-37 | 1.53256E+36 | 5.77825E-37 | 1 |
| Fam+R.Temp+F.Temp | 4456.46 | 174.04 | 1.61322E-38 | 6.19877E+37 | 1.42859E-38 | 1 |
| R.Temp+F.Temp+R.Temp:F.Temp | 4507.62 | 225.20 | 1.25442E-49 | 7.97184E+48 | 1.11085E-49 | 1 |
| Pop+Fam[Pop]&Random+F.Temp+ Pop:F.Temp | 4507.89 | 225.47 | 1.096E-49 | 9.12407E+48 | 9.70563E-50 | 1 |
| An+Pop[An]&Random+Fam[An,Pop]&Random+F.Temp+An:F.Temp | 4515.41 | 232.99 | 2.5519E-51 | 3.91864E+50 | 2.25983E-51 | 1 |
| R.Temp+F.Temp | 4516.70 | 234.28 | 1.33889E-51 | 7.46888E+50 | 1.18565E-51 | 1 |
| Pop+Fam[Pop]&Random+F.Temp | 4529.19 | 246.77 | 2.59762E-54 | 3.84968E+53 | 2.30032E-54 | 1 |
| An+Pop[An]&Random+Fam[An,Pop]&Random+F.Temp | 4531.45 | 249.03 | 8.39117E-55 | 1.19173E+54 | 7.43079E-55 | 1 |
| Pop+Fam[Pop]&Random+R.Temp+ Pop:R.Temp | 4551.04 | 268.62 | 4.67636E-59 | 2.13841E+58 | 4.14114E-59 | 1 |
| Pop+Fam[Pop]&Random+R.Temp | 4556.52 | 274.10 | 3.01954E-60 | 3.31176E+59 | 2.67395E-60 | 1 |
| An+Pop[An]&Random+Fam[An,Pop]&Random+R.Temp+An:R.Temp | 4558.48 | 276.06 | 1.13327E-60 | 8.82404E+59 | 1.00356E-60 | 1 |
| An+Pop[An]&Random+Fam[An,Pop]&Random+R.Temp | 4560.75 | 278.33 | 3.64257E-61 | 2.74531E+60 | 3.22568E-61 | 1 |
| Fam+F.Temp | 4566.02 | 283.60 | 2.61242E-62 | 3.82787E+61 | 2.31342E-62 | 1 |
| Fam+F.Temp+Fam:F.Temp | 4586.31 | 303.89 | 1.02595E-66 | 9.74709E+65 | 9.08526E-67 | 1 |
| F.Temp | 4592.17 | 309.75 | 5.47825E-68 | 1.8254E+67 | 4.85126E-68 | 1 |
| Fam+R.Temp | 4595.10 | 312.68 | 1.2659E-68 | 7.89949E+67 | 1.12102E-68 | 1 |
| Pop+Fam[Pop]&Random | 4618.67 | 336.25 | 9.64362E-74 | 1.03695E+73 | 8.53989E-74 | 1 |
| R.Temp | 4619.76 | 337.34 | 5.59178E-74 | 1.78834E+73 | 4.95179E-74 | 1 |
| An+Pop[An]&Random+Fam[An,Pop]&Random | 4622.14 | 339.72 | 1.70114E-74 | 5.87842E+73 | 1.50644E-74 | 1 |
| Fam+R.Temp+Fam:R.Temp | 4630.57 | 348.15 | 2.51297E-76 | 3.97935E+75 | 2.22536E-76 | 1 |
| Fam | 4658.42 | 376.00 | 2.25236E-82 | 4.43979E+81 | 1.99457E-82 | 1 |
|  |  | Sum: | 1.129243769 |  |  |  |

**(D) RMR of fry (population nested within ancestry; family not included as a predictive variable)**

| **Model** | **AICc** | **delta(i)= di** | **exp(-0.5*di)** | **ER** | **wi** | **Additive wi** |
| --- | --- | --- | --- | --- | --- | --- |
|  |  |  |  |  |  |  |
| **Pop+Mass+F.Temp+ Pop:F.Temp** | **4293.04** | **0.00** | **1** | **1** | **0.756505166** | **0.756505166** |
| *Pop+Mass+R.Temp+F.Temp+ R.Temp:F.Temp* | *4295.5* | *2.46* | *0.292292578* | *3.421229536* | *0.221120845* | *0.977626011* |
| An+Pop[An]&Random+Mass+ R.Temp+F.Temp+R.Temp:F.Temp | 4301.66 | 8.62 | 0.01343355 | 74.44048894 | 0.01016255 | 0.987788561 |
| Pop+Mass+F.Temp | 4302.18 | 9.14 | 0.01035796 | 96.54410977 | 0.00783585 | 0.995624411 |
| Pop+Mass+R.Temp+F.Temp | 4303.51 | 10.47 | 0.005326824 | 187.7291063 | 0.00402977 | 0.999654181 |
| An+Pop[An]&Random+Mass+ F.Temp | 4309.71 | 16.67 | 0.000239969 | 4167.201557 | 0.000181538 | 0.999835719 |
| An+Pop[An]&Random+Mass+ R.Temp+F.Temp | 4309.92 | 16.88 | 0.00021605 | 4628.554985 | 0.000163443 | 0.999999162 |
| Mass+F.Temp+Mass:F.Temp | 4321.51 | 28.47 | 6.57382E-07 | 1521184.704 | 4.97313E-07 | 0.999999659 |
| Mass+R.Temp+F.Temp+ R.Temp:F.Temp | 4322.27 | 29.23 | 4.49558E-07 | 2224404.951 | 3.40093E-07 | 0.999999999 |
| Mass+R.Temp+F.Temp | 4334.56 | 41.52 | 9.63932E-10 | 1037417200 | 7.2922E-10 | 1 |
| Mass+F.Temp | 4339.00 | 45.96 | 1.04692E-10 | 9551843410 | 7.91999E-11 | 1 |
| Pop+Mass+R.Temp+Pop:R.Temp | 4375.37 | 82.33 | 1.32516E-18 | 7.54627E+17 | 1.00249E-18 | 1 |
| Pop+Mass+R.Temp | 4377.74 | 84.70 | 4.05162E-19 | 2.46815E+18 | 3.06507E-19 | 1 |
| Pop+Mass+Pop:Mass | 4381.87 | 88.83 | 5.1382E-20 | 1.94621E+19 | 3.88707E-20 | 1 |
| An+Pop[An]&Random+Mass+ R.Temp | 4384.02 | 90.98 | 1.75365E-20 | 5.70238E+19 | 1.32665E-20 | 1 |
| Pop+Mass | 4390.01 | 96.97 | 8.7747E-22 | 1.13964E+21 | 6.6381E-22 | 1 |
| Mass+R.Temp | 4393.71 | 100.67 | 1.37971E-22 | 7.24791E+21 | 1.04376E-22 | 1 |
| Mass+R.Temp+Mass:R.Temp | 4394.24 | 101.20 | 1.05852E-22 | 9.44715E+21 | 8.00776E-23 | 1 |
| An+Pop[An]&Random+Mass | 4397 | 103.96 | 2.66301E-23 | 3.75515E+22 | 2.01458E-23 | 1 |
| An+Pop[An]&Random+Mass+ An:Mass | 4406.52 | 113.48 | 2.28103E-25 | 4.38398E+24 | 1.72561E-25 | 1 |
| Mass | 4413.65 | 120.61 | 6.45463E-27 | 1.54928E+26 | 4.88296E-27 | 1 |
| An+Pop[An]&Random+R.Temp+ F.Temp+R.Temp:F.Temp | 4426.89 | 133.85 | 8.6068E-30 | 1.16187E+29 | 6.51109E-30 | 1 |
| Pop+R.Temp+F.Temp+ R.Temp:F.Temp | 4430.71 | 137.67 | 1.2745E-30 | 7.84622E+29 | 9.64165E-31 | 1 |
| Pop+R.Temp+F.Temp+ R.Temp:F.Temp | 4430.71 | 137.67 | 1.2745E-30 | 7.84622E+29 | 9.64165E-31 | 1 |
| An+Pop[An]&Random+R.Temp+ F.Temp | 4433.26 | 140.22 | 3.56134E-31 | 2.80793E+30 | 2.69417E-31 | 1 |
| Pop+R.Temp+F.Temp | 4437.13 | 144.09 | 5.14344E-32 | 1.94422E+31 | 3.89104E-32 | 1 |
| R.Temp+F.Temp+R.Temp:F.Temp | 4507.62 | 214.58 | 2.53831E-47 | 3.93963E+46 | 1.92025E-47 | 1 |
| An+Pop[An]&Random+F.Temp+ An:F.Temp | 4518.97 | 225.93 | 8.70811E-50 | 1.14835E+49 | 6.58773E-50 | 1 |
| Pop+F.Temp+Pop:F.Temp | 4528.90 | 235.86 | 6.07648E-52 | 1.64569E+51 | 4.59689E-52 | 1 |
| An+Pop[An]&Random+F.Temp | 4532.28 | 239.24 | 1.12123E-52 | 8.91879E+51 | 8.48215E-53 | 1 |
| Pop+F.Temp | 4540.98 | 247.94 | 1.44715E-54 | 6.91014E+53 | 1.09478E-54 | 1 |
| An+Pop[An]&Random+R.Temp | 4561.73 | 268.69 | 4.51552E-59 | 2.21458E+58 | 3.41601E-59 | 1 |
| An+Pop[An]&Random+R.Temp+ An:R.Temp | 4562.26 | 269.22 | 3.46433E-59 | 2.88656E+58 | 2.62079E-59 | 1 |
| Pop+R.Temp | 4568.68 | 275.64 | 1.39809E-60 | 7.15263E+59 | 1.05766E-60 | 1 |
| Pop+R.Temp+Pop:R.Temp | 4573.02 | 279.98 | 1.5963E-61 | 6.26447E+60 | 1.20761E-61 | 1 |
| F.Temp | 4592.17 | 299.13 | 1.10853E-65 | 9.02099E+64 | 8.38606E-66 | 1 |
| R.Temp | 4619.76 | 326.72 | 1.1315E-71 | 8.83785E+70 | 8.55983E-72 | 1 |
| An+Pop[An]&Random | 4620.75 | 327.71 | 6.89728E-72 | 1.44985E+71 | 5.21783E-72 | 1 |
| Pop | 4629.62 | 336.58 | 8.17677E-74 | 1.22298E+73 | 6.18577E-74 | 1 |
|  |  | Sum: | 1.321868039 |  |  |  |
